# Differentiating *Huangjiu* with Varying Sugar Contents from Different Regions Based on Targeted Metabolomics Analyses of Volatile Carbonyl Compounds

**DOI:** 10.3390/foods12071455

**Published:** 2023-03-29

**Authors:** Junting Yu, Zhilei Zhou, Xibiao Xu, Huan Ren, Min Gong, Zhongwei Ji, Shuangping Liu, Zhiming Hu, Jian Mao

**Affiliations:** 1 National Engineering Research Center of Cereal Fermentation and Food Biomanufacturing, School of Food Science and Technology, Jiangnan University, Wuxi 214122, China; 2 Jiangnan University (Shaoxing) Industrial Technology Research Institute, Shaoxing 312000, China; 3 Jiangsu Provincial Engineering Research Center for Bioactive Product Processing, Jiangnan University, Wuxi 214122, China; 4 Shaoxing Nverhong Winery Co., Ltd., Shaoxing 312000, China; 5 College of Life Sciences, Linyi University, Linyi 276000, China; 6 National Engineering Research Center for *Huangjiu*, Shaoxing 312000, China

**Keywords:** *Huangjiu*, carbonyl compounds, PFBHA, targeted metabolomics, OPLS-DA

## Abstract

*Huangjiu* is one of the oldest alcoholic beverages in the world. It is usually made by fermenting grains, and Qu is used as a saccharifying and fermenting agent. In this study, we identified differential carbonyl compounds in *Huangjiu* with varying sugar contents from different regions. First, we developed and validated a detection method for volatile carbonyl compounds in *Huangjiu*, and for optimal extraction, 5 mL of *Huangjiu* and 1.3 g/L of O-(2,3,4,5,6-pentafluorobenzyl)hydroxylamine hydrochloride (PFBHA) were incubated at 45 °C for 5 min before extracting the volatile carbonyl compounds at 45 °C for 35 min. Second, the targeted quantitative analysis of 50 carbonyl compounds in *Huangjiu* showed high levels of Strecker aldehydes and furans. Finally, orthogonal projections to latent structures discriminant analysis (OPLS-DA) was used to differentiate between *Huangjiu* with different sugar contents, raw materials, and region of origin. A total of 19 differential carbonyl compounds (VIP > 1, *p* < 0.05) were found in *Huangjiu* with different sugar contents (semidry and semisweet *Huangjiu*), and 20 differential carbonyl compounds (VIP > 1, *p* < 0.05) were found in different raw materials for *Huangjiu* production (rice and nonrice *Huangjiu*). A total of twenty-two and eight differential carbonyl compounds, with VIP > 1 and *p* < 0.05, were identified in semidry and semisweet *Huangjiu* from different regions (Zhejiang, Jiangsu, Shanghai, and Fujian), respectively.

## 1. Introduction

**Figure 1:**
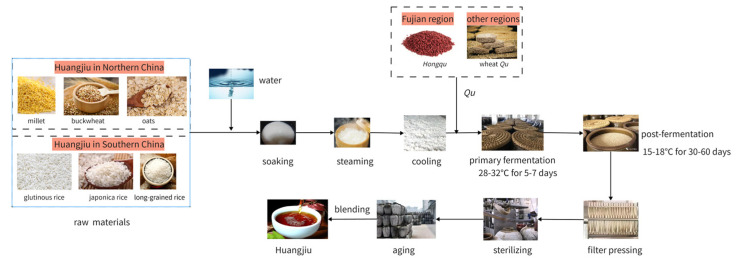
The traditional production process of *Huangjiu*.

Chinese rice wine (*Huangjiu)*, along with beer and wine, is one of the oldest alcoholic beverages in the world [1]. *Huangjiu* is usually made by fermenting grains, with Qu as a saccharifying and fermenting agent. The traditional process of *Huangjiu* production generally includes maceration, steaming of materials, cooling and addition of Qu, primary fermentation, Kaipa, post-fermentation, pressing and filtering, boiling and sterilization, storage, blending, and packaging [2], as shown in Figure 1. Owing to its specific brewing process, the flavor profile of *Huangjiu* differs markedly from those of beer, wine, and other alcoholic beverages [1]. Depending on the region and the primary raw materials used, *Huangjiu* can be divided into the following two categories: rice *Huangjiu*, which is typically made with glutinous rice, with japonica rice as the primary raw material, and is primarily produced in southern China, and nonrice *Huangjiu*, which is typically made with millet, buckwheat, and oats as the primary raw materials and is primarily produced in northern China [3]. Furthermore, depending on the total sugar contents, *Huangjiu* can be divided into dry *Huangjiu* (≤15 g/L), semidry *Huangjiu* (15.1–40.0 g/L), semisweet *Huangjiu* (40.1–100 g/L), and sweet *Huangjiu* (>100 g/L) [4].

Levels of carbonyl compounds are usually low in alcoholic beverages, but being the diverse and important class of flavoring substances, they are common in wine [5,6], beer [7,8,9], vodka [10], brandy [11], and Chinese baijiu [12,13]. Some carbonyl compounds are directly derived from raw materials, whereas others are produced during alcoholic fermentation and aging processes through a series of chemical reactions, such as oxidation of fatty acids and higher alcohols, Strecker degradation, aldehyde condensation, or the Maillard reactions [9,14,15,16,17,18,19]; thus, carbonyl compounds are susceptible to various factors and can be used as the key to differentiate among beverages. Carbonyl compounds are highly reactive compounds [20], which are difficult to trap by Headspace Solid-Phase Microextraction (HS-SPME) owing to their low content in *Huangjiu* and their volatile and reactive nature; however, the properties of these compounds can be changed by derivatization to be more favorable for SPME. In many previous studies, PFBHA was used for the detection and derivatization of carbonyl compounds in alcoholic beverages [5,8,9,21].

Metabolomics has been widely used in recent years for the identification and differentiation of compounds of food origins. Peng Q. et al. used gas chromatography (GC)-flash electronic nose in combination with chemometrics to successfully differentiate between Chinese Tongshan kaoliang spirits from different geographical origins [22]. Leborgne C. et al. reported the color of rosé wines by polyphenol-targeted metabolomics, which can also be applied to identify the varietal and geographical origin of the wines [23]. Moreira N et al. used PFBHA derivatization combined with HS-SPME-GC-MS to determine the content of carbonyl compounds in various aged port wines, and to differentiate among them [5]. However, there are no studies on targeted metabolomics using GC-MS to analyze volatile carbonyl compounds in *Huangjiu*.

In this study, we aimed to develop and establish a method for the analysis of volatile carbonyl compounds in *Huangjiu* by PFBHA derivatization combined with HS-SPME-GC-MS, and to investigate the differences in volatile carbonyl compounds contents in *Huangjiu* from different regions and varying sugar contents using targeted quantitative metabolomics and multivariate statistical analysis.

## 2. Materials and Methods

### 2.1. Samples

A total of 55 commercial *Huangjiu* samples were used in this study, which were divided into 27 semidry and 28 semisweet *Huangjiu* according to their sugar content, and into rice and nonrice *Huangjiu* according to the raw materials used. Among them, rice-semidry *Huangjiu* included six *Huangjiu* of Jiangsu origin (SDJ), nine of Zhejiang origin (SDZ), four of Shanghai origin (SDS), and eight of Fujian origin (SDF). Rice-semisweet *Huangjiu* included four *Huangjiu* of Jiangsu origin (SSJ), six of Zhejiang origin (SSZ), four of Shanghai origin (SSS), and three of Fujian origin (SSF). Nonrice-semisweet *Huangjiu* were 11 in number and from various origins (SSN).

### 2.2. Reagents and Standards

PFBHA (≥99%), hexanal (≥96%), nonanal (≥95%), decanal (≥97%), 2-methylpropanal (≥99%), (E)-2-heptenal (≥95%), glyoxal (≥40%), phenyl acetaldehyde (≥95%), furfural (≥99%), 5-methyl-2-furfural (≥95%), methional (≥95%), 1-octen-3-one (≥97%), and cyclopentanone (≥99%) were purchased from J&K Scientific (Beijing, China). Ethanal (≥99.5%), propanal (≥99%), heptanal (≥98%), and acrolein (≥99%) were purchased from Aladdin (Shanghai, China). Butanal (≥99.5%), pentanal (≥98%), undecanal (≥97%), dodecanal (≥95%), tridecanal (≥95%), (E)-2-butenal (≥98%), (E)-2-pentenal (≥95%), (E)-2-hexenal (≥98%), (E)-2-octenal (≥95%), (E)-2-nonenal (≥95%), (E)-2-decenal (≥95%), 2-pentanone (≥99%), 2-hexanone (≥99%), 4-heptanone (≥98%), 2-heptanone (≥98%), 2-nonanone (≥99%), benzaldehyde (≥99%), 2-phenyl-2-butenal (≥97%), 3-methyl-2-butanone (≥99.5%), 3-pentanone (≥99.5%), 3-penten-2-one (≥95%), and cyclohexanone (≥99.7%) were purchased from Macklin (Shanghai, China). Acetophenone (≥99.5%), 2-methylbutanal (≥98%), 3-methylbutanal (≥98%), 2-octanone (≥99%), methyl glyoxal (≥40%), 6-methyl-5-hepten-2-one (≥97%), and ethanol (≥99.8%) were obtained from Sinopharm Chemical Reagent (Shanghai, China). Octanal (≥98%), 2-decanone (≥99%), acetoin (≥98%), diacetyl (≥98%), and 3-methyl-2-butenal (≥97%) were purchased from TCI (Shanghai, China). *p*-fluorobenzaldehyde (internal standard, ≥98%) was purchased from Sigma-Aldrich (Shanghai, China).

Ultrapure water was obtained from a Milli-Q water purification system (Millipore, France). The divinylbenzene/carbon wide range/polydimethylsiloxane (DVB/CWR/PDMS, 80 μm) SPME fibers were purchased from Agilent Technologies (Geneva, Switzerland).

The 20 g/L PFBHA solution was prepared with ultrapure water on a daily basis. A 107 mg/L internal standard (*p*-fluorobenzaldehyde) solution was prepared in ethanol. Standard solutions of the other compounds were also prepared in ethanol and diluted with a *Huangjiu* simulant (15% ethanol, 5 g/L lactic acid, pH = 4). All solutions were stored at 4 °C.

### 2.3. Derivatization and HS-SPME Procedure

Based on the derivatization method of Nathalie Moreira et al. [5], HS-SPME-related experimental parameters were optimized. The experiment was performed with a DVB/CWR/PDMS (80 μm) extraction head (Agilent Technologies, Geneva, Switzerland), and the HS-SPME procedure was performed with a Combi-PAL autosampler (Varian Pal Autosampler, Geneva, Switzerland). After equilibrating the vial at the extraction temperature for 3–15 min with stirring at 250 rpm, the extraction head was exposed to HS at the same temperature, and the extraction head was desorbed at the GC injector for 5 min.

First, the effect of sample volume on the extraction efficiency was evaluated by adding 2, 3, 4, and 5 mL of *Huangjiu*, 5 μL of internal standard (107 mg/L), and 2 g/L of PFBHA (20 g/L) in a 20 mL vial. After incubating at 50 °C for 5 min, extraction was performed at 50 °C for 50 min. Next, using the same extraction procedures and adding 0, 1, and 2 g of NaCl to the vials containing 5 mL of *Huangjiu* samples, the effect of the ionic strength on the extraction efficiency was assessed.

The experimental parameters, including extraction temperature, extraction time, incubation time, and PFBHA concentration were optimized by a central composite design (CCD, α = 2.000) including a 2^4^ factorial design with eight axial points and five replicates in the center of the design. A total of 29 experiments were generated by CCD and executed in random order. The factor levels and experimental domains are listed in Table 1. CCD experiments allowed for a reduction in the number of experiments, thereby reducing time and cost, while assessing the contribution of each parameter and the relationship among them [5,24].

### 2.4. GC-MS Analysis

An Agilent 8890 GC (Agilent Technologies, Shanghai, China) and a 7000D triple quadrupole MS were employed, and VF-WAXms (30 m × 0.25 mm × 0.25 μm, Agilent Technologies, Amsterdam, Netherlands) columns were used. The injection port temperature was 250 °C in a nonsplit injection mode. High-purity helium (≥99.999%) was used as the carrier gas at a constant flow rate of 1 mL/min. The initial oven temperature was held for 2 min at 40 °C, which increased up to 230 °C at a rate of 5 °C/min and was held for 10 min. Electron ionization (EI) mode was used at 70 eV ionization energy. The ion source temperature was 230 °C. The characterization was performed in full scan mode, 33–400 *m*/*z*, at a scan rate of 6 scan/s. The quantitative analysis of carbonyl compounds was performed in the selected ion monitoring mode.

### 2.5. Qualitative and Quantitative Analyses

After PFBHA derivatization, most carbonyl compounds generated two or more isomers [24], and the identification results are shown in Table 2. The mass spectra of PFBHA derivatives with 181 *m*/*z* as the characteristic ion were used to characterize the compounds by (1) comparing MS ion fragments with the National Institute of Standards and Technology (NIST 20) with >70% similarity; (2) comparing the retention time and mass spectra of the standard compounds under the same experimental conditions; and (3) the retention indices of each compound were calculated by n-alkanes (C7–C30) and identified by comparing the retention indices of the standard compounds with those described in the relevant literature.

The carbonyl compounds were quantified by establishing a standard curve, which was carried out in a simulant *Huangjiu* (15% ethanol, 5 g/L lactic acid, pH = 4). If isomers were present after PFBHA derivatization, the sum of the isomer peak areas was used for quantification.

### 2.6. Method Validation

The limit of detection (LOD) was defined as the minimum concentration of the substance that can be detected when the signal-to-noise ratio is 3, and the limit of quantification (LOQ) was defined as the minimum concentration of the substance that can be quantified when the signal-to-noise ratio is 10. The precision experiment included interday and intraday precision. In the intraday precision experiment, six samples of the same level were measured on the same day, whereas in the interday precision test, six samples of the same level were analyzed on different days, and the precision results are expressed as the relative standard deviation (RSD).

Referring to the method in the literature [25], the volatile compounds in *Huangjiu* were removed by solvent-assisted flavor evaporation. The nonvolatile matrix was adjusted to the initial volume with 15% ethanol and was extracted twice with Poly-Sery PSD cartridges (500 mg, 6 mL/30 pcs, CNW, Shanghai, China) to remove the residual volatiles until the *Huangjiu* was odorless. The recoveries were determined by adding standard solutions of carbonyl compounds at low, medium, and high concentrations to the odorless *Huangjiu*.

### 2.7. Statistical Analyses

All *Huangjiu* samples were obtained in triplicate, and results are expressed as mean ± standard deviation (Mean ± SD). The CCD design and analysis were performed using Statistica 12 (Statsoft Inc., Tulsa, OK, USA). Analysis of variance (ANOVA) was performed to analyze the data using SPSS 26 (SPSS Inc., Chicago, IL, USA). OPLS-DA was performed with SIMCA-P 13 (Umetrics, Umea, Sweden). GraphPad Prism8 (GraphPad Software, CA, USA) software was used to perform the Student’s *t*-test, and differences were deemed significant at *p* < 0.05.

## 3. Results and Discussion

### 3.1. Extraction Method Optimization

First, the effect of sample volume on the derivative extraction effect was determined, and the results showed that the volume of the sample did not significantly affect the extraction effect, which is consistent with previous findings in alcoholic beverages (Figure S1) [5]. Therefore, the sample addition volume was kept at 5 mL. Secondly, NaCl of different concentrations was used to determine the effect of ionic strength on the extraction effect, which frequently affected the extraction of volatiles because of the salt precipitation effect (Figure S2). Under the experimental conditions, the extraction efficiency of the samples with the addition of NaCl decreased significantly, which is consistent with the results of previous studies [9,21]. This indicated that the addition of NaCl increased the spatial potential resistance effect and decreased the extraction efficiency; therefore, the samples were prepared without the addition of NaCl.

HS-SPME conditions and PFBHA addition levels were optimized using a CCD. The response values were based on the total peak areas of carbonyl compound derivatives, and some substances with high contents, such as ethanal, 2-butanone, 2-methylpropanal, 3-methylbutanal, acetoin, 2-furfural, and benzaldehyde, were excluded from the total peak areas to prevent their interference with the results. Thus, a good response model was constructed. The results are shown in Table S1. ANOVA was performed to determine the importance of each experimental factor and its interactions, which yielded a coefficient of determination R^2^ of 0.986 and an adjusted-R^2^ of 0.973 for the method, indicating that the experimental data had a good fit. The experimental error was calculated using replication values of the central point with an RSD of 2.80%, indicating that the method had good reproducibility. More details of ANOVA are shown in Table S2. The multivariate quadratic corresponding surface regression model was obtained, as given:

Total area = −2.442 × 10^8^ − 3.254 × 10^6^ × A + 2.372 × 10^8^ × B + 3.006 × 10^6^ × C + 8.637 × 10^6^ × D − 8.890 × 10^5^ × AB − 1.226 × 10^5^ × AC − 8.881 × 10^4^ ×AD + 2.045 × 10^6^× BC − 3.347 × 10^6^ × BD + 1.065 × 10^5^ × CD + 5.602 × 10^5^ × A2 − 7.498 × 10^7^ × B2 − 3.107 × 10^4^ × C2 − 5.599× 10^4^ × D2

**Figure 2:**
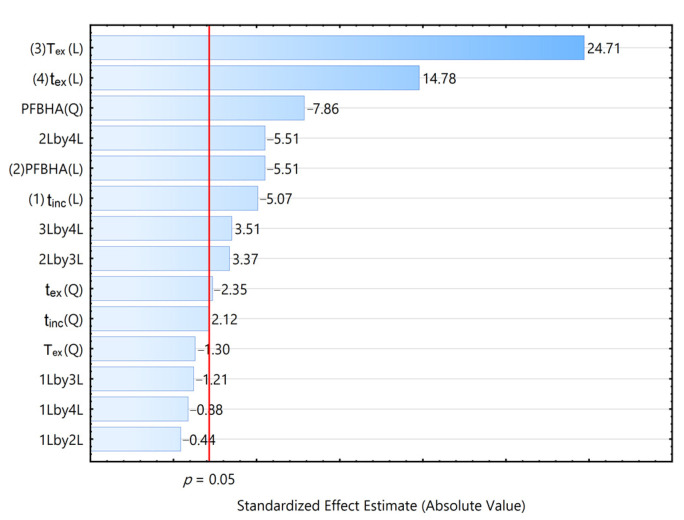
Pareto chart for the total area of all volatile carbonyl compounds of the GC–MS analysis of the HS-SPME of *Huangjiu*.

To characterize the primary factors and their interactions that affect the extraction efficiency, a Pareto chart was used (Figure 2). Extraction temperature and time were found to have a strong positive effect on the results, whereas PFBHA concentration and incubation time were found to have a negative effect on the results. The extraction temperature is a crucial factor affecting the derivative extraction of carbonyl compounds, which affects the partition coefficients of volatile compounds between the sample and HS, and between HS and the extracted fiber head. The increase in extraction temperature increases the HS concentration of volatile compounds, but decreases the adsorption of top air to the fiber [21,26]. The high concentration of PFBHA saturates the extraction fiber head with PFBHA, thereby decreasing the total amount of volatile carbonyl compounds analyzed [8].

**Figure 3:**
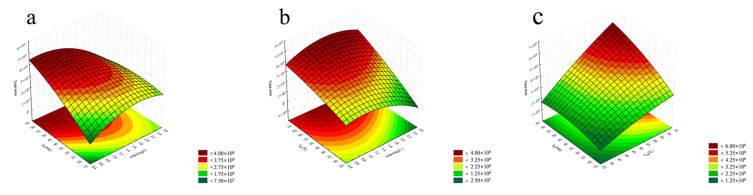
Response surface model for the total area of all volatile compounds vs. (**a**) extraction time (t_ex_) and PFBHA content (incubation time = 9 min, temperature = 50 °C), (**b**) extraction temperature (T_ex_) and PFBHA content (incubation time = 9 min, extraction time = 40 min), and (**c**) extraction temperature (T_ex_) and extraction time (t_ex_) (incubation time = 9 min, PFBHA = 1.5 g/L).

A significant interaction was observed between the experimental factors affecting the response values of carbonyl compound derivatives. The interaction between extraction time and PFBHA concentration showed that the response values were high at low PFBHA concentrations (Figure 3a). The interaction between PFBHA and extraction temperature resulted in greater response values at higher temperatures (Figure 3b). Additionally, the interaction between extraction time and extraction temperature significantly affected the extraction results, with the response values increasing significantly with the increase in temperature and time (Figure 3c).

Incubation time has a negative effect on extraction efficiency, but according to past experience, this time cannot be ignored, and a smaller reaction time of 5 min is optional [21]. There is an interaction between extraction time, PFBHA, and extraction temperature. In order to improve experimental efficiency, the extraction time was less than 40 min. Too high a temperature can denature *Huangjiu*, so the temperature should not be too high, ranging from 40 to 50 °C. For the convenience of the experiment, the extraction temperature and extraction time should preferably be integers. The concentration of PFBHA is greatly affected by temperature and time, and the overall impact on extraction efficiency increases first, and then decreases. Based on the above considerations, the optimal experimental parameters can be obtained according to the corresponding optimizer, as shown in Figure S3.

Therefore, the optimal experimental conditions for the analysis of volatile carbonyl compounds in *Huangjiu* were as follows: PFBHA concentration of 1.3 g/L, extraction temperature of 45 °C for 35 min, and SPME fiber addition after 5 min of incubation. Comparison with the optimal parameters for carbonyl compound extraction in port wine [5] and beer [8] showed that the optimal experimental conditions for the derivative extraction of carbonyl compounds in different alcoholic beverages differed, owing to the differences in the matrix.

### 3.2. Method Validation

The linear range, coefficient of determination, LOD, and LOQ of the method were determined under optimal experimental conditions, and the results are shown in Table 3. A total of 49 carbonyl compounds, including 11 saturated aldehydes, 8 alkenes, 7 Strecker aldehydes, 3 dicarbonyl compounds, 9 ketones, 2 furans, and 9 other carbonyl compounds were included, with good linearity (r^2^ ≥ 0.996). The LOD of the compounds ranged from 0.002 to 0.105 μg/L, whereas the LOQ of the compound ranged from 0.007 to 0.317 μg/L. As shown in Table S3, the developed method had a good precision with RSD < 10% for both interday and intraday precision. Furthermore, the recoveries obtained by the method ranged from 91% to 109%, indicating the high accuracy of the method. Therefore, the developed method is suitable for the determination of carbonyl compounds in *Huangjiu*.

### 3.3. Quantification of Volatile Carbonyl Compounds in Huangjiu

The volatile carbonyl compounds in *Huangjiu* were qualitatively analyzed, and the results are shown in Table 2. A total of 53 carbonyl compounds were identified, including 12 alkanals, 8 alkenals, 7 Strecker aldehydes, 3 dicarbonyl compounds, 11 ketones, 3 furans, and 9 other carbonyl compounds, among which glyoxal and methyl glyoxal have not been reported in previous studies on *Huangjiu*. No significant difference was found in the number of carbonyl compounds found in *Huangjiu* of different origins and with sugar content.

The 50 carbonyl compounds of semidry *Huangjiu* (Table S4) and semisweet *Huangjiu* (Table S5) were quantitatively analyzed. The results showed that *Huangjiu* contained high levels of propanal, pentanal, hexanal, (E)-2-butenal, 2-methylpropanal, 2-methylbutanal, 3-methylbutanal, benzaldehyde, phenyl acetaldehyde, diacetyl, glyoxal, methyl glyoxal, 2-butanone, 2-furfural, 5-methyl-2-furfural, 3-penten-2-one, acetoin, and 2-phenyl-2-butenal. Among them, alkanals might be obtained from the oxidative degradation of fatty acids, such as enzymatic oxidation of linoleic/linolenic acid, and hexanal is the main product of linoleic acid oxidation [27]. Valine, isoleucine, and leucine yielded 2-methylpropanal, 2-methylbutanal, and 3-methylbutanal, respectively, through Strecker degradation [20]. The oxidation of benzyl alcohol and the action of microbes on aromatic amino acids, phenylacetic acid, and p-hydroxybenzoic acid can all result in the production of benzaldehyde [28], and its concentration can increase with the aging process [29]. Diacetyl can be produced by thermal degradation of the Maillard reactions [20], and a significant difference was found between fresh and aged *Huangjiu* [30]. Furans (2-furfural, 5-methyl-2-furfural) have a typical caramel aroma, which can be used to distinguish between fresh and aged *Huangjiu* [30,31], as its content increases during the aging process [1]. Furans are the thermal degradation product of the Maillard reaction [20]. The compound 2-Phenyl-2-butenal is produced during the aging process of *Huangjiu* and is a key substance [32,33]. Acetoin has a milky aroma and is a crucial component of *Huangjiu* [34].

The differences in the contents of carbonyl compounds in semidry and semisweet *Huangjiu* from different origins were analyzed. The contents of propanal, pentanal, heptanal, undecanal, methyl glyoxal, and 2-cyclohexen-1-one in semidry *Huangjiu* from the Fujian region were significantly higher than those in the other three regions, probably because it is *HongquHuangjiu,* and the saccharifying ferments used are different from those in other regions [35,36]. The contents of dodecanal, butenal, 3-methyl-2-butenal, glyoxal, 2-pentanone, and cyclopentanone in *Huangjiu* from the Shanghai region were significantly higher than those from other regions. Shanghai *Huangjiu* has the same root as Zhejiang *Huangjiu*, but Shanghai *Huangjiu* introduced filtration and canning processes in the brewing process, and also added honey and wolfberry, which improved the bitterness of traditional Zhejiang *Huangjiu* and made the flavor composition very different. The content of 2-heptanone in *Huangjiu* from the Jiangsu region is significantly lower than that of *Huangjiu* from other regions, and the content of methional and acetoin is significantly higher than that of *Huangjiu* from other regions, probably due to the fact that *Huangjiu* from other regions mainly uses glutinous rice as raw material, while *Huangjiu* from Jiangsu uses japonica rice instead of glutinous rice [37]. Glutinous rice is the best rice for making *Huangjiu* [38]; glutinous rice has a higher content of branched chain starch, which is more easily pasted and digested by enzymes [39], and is the main reason for the mellow and pleasant taste of *Huangjiu*. Compared to glutinous rice, japonica rice has a higher protein content and produces a more nutritious *Huangjiu* with a higher amino acid content [40]. Among them, Yangzhou rice (japonica rice) is considered to be a good substitute for glutinous rice in the *Huangjiu* brewing process [41]. The content of 2-phenyl-2-butenal in *Huangjiu* from the Zhejiang region was significantly higher than that of other regions, in agreement with the results of Xiao et al. [33] when they studied the classification of *Huangjiu* by region.

Nonrice-semisweet *Huangjiu* contained significantly higher levels of (E)-2-heptenal and significantly lower levels of 3-methylbutanal than rice *Huangjiu*. The main raw materials of rice *Huangjiu* are glutinous rice and japonica rice, which are produced in Jiangsu, Zhejiang, Shanghai, Fujian, and other southern regions of China, while the main raw materials of nonrice *Huangjiu* are millet, corn, and other coarse grains, produced in Shandong, Shanxi, and other northern regions of China [42]. Because there is a big difference in the nutritional composition of millet and rice, the quality and flavor of the *Huangjiu* produced also differs greatly [40].

### 3.4. OPLS-DA Distinguishes between Carbonyl Compounds in Different Huangjiu

The content of carbonyl compounds in *Huangjiu* from different regions and with different sugar contents varies, which may be because of raw materials [39,41], ferments [36], brewing processes, and aging time [43,44]; however, *Huangjiu* has strong regional characteristics in terms of flavor composition.

#### 3.4.1. OPLS-DA of Semidry and Semisweet *Huangjiu*

**Figure 4:**
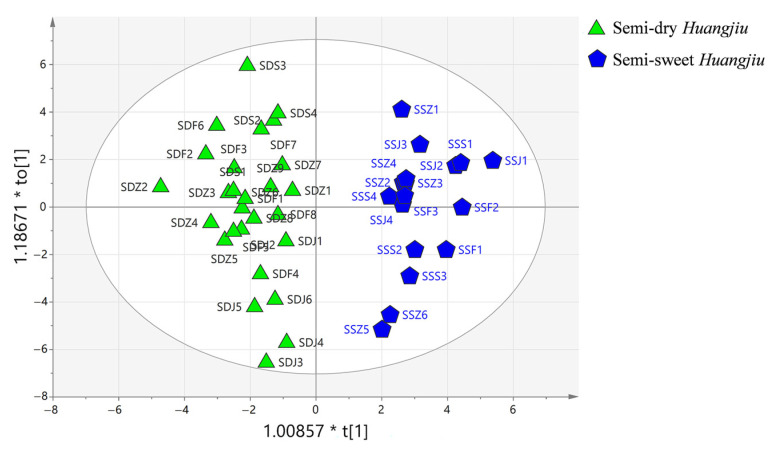
OPLS-DA of semidry *Huangjiu* and semisweet *Huangjiu*.

Data on carbonyl compounds in two sweet types of *Huangjiu*, semidry and semisweet, were modeled and analyzed (Figure 4) using rice *Huangjiu* samples. Figure 4 shows clear separation (R^2^Y = 0.885), and the model exhibited good predictive power (Q^2^ = 0.813). Based on the OPLS-DA, a total of 19 volatile carbonyl compounds with VIP > 1 and *p* < 0.05 were screened, and the results are shown in Table 4. The difference between semidry *Huangjiu* and semisweet *Huangjiu* lies firstly in the brewing process. Semidry *Huangjiu* is brewed with water, whereas semisweet *Huangjiu* is brewed with finished *Huangjiu* instead of water, thus causing differences in sugars and flavors between the two types of *Huangjiu*, which may also be the reason for the different contents of carbonyl compounds. The generally higher concentration of carbonyl compounds in semidry *Huangjiu*, particularly with more benzaldehyde, 2-phenyl-2-butenal, 3-methylbutanal, 2-methylbutanal, and 2-furfural, may be the cause of the stronger and more complex aroma [4].

#### 3.4.2. OPLS-DA of Rice *Huangjiu* and Nonrice *Huangjiu*

**Figure 5:**
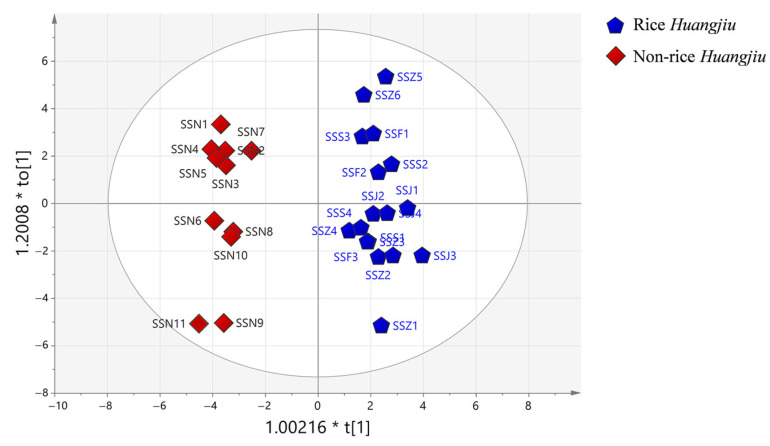
OPLS-DA of rice *Huangjiu* and nonrice *Huangjiu*.

The OPLS-DA based on carbonyl compounds in different raw *Huangjiu* showed significant differences between rice *Huangjiu* and nonrice *Huangjiu* with clear separation and good predictive power (R^2^Y = 0.958, Q^2^ = 0.811, Figure 5), and a total of 20 carbonyl compounds with VIP > 1 and *p* < 0.05 were identified (Table 5). Rice *Huangjiu* contained higher levels of Strecker aldehydes (3-methylbutanal, benzaldehyde, 2-methylpropanal, and 2-methylbutanal) than nonrice *Huangjiu*. The content of Strecker aldehydes is strongly correlated to the aging time [29]. Thus, the higher content of Strecker aldehydes in rice *Huangjiu* may be because of different raw materials, or because of a longer aging time than that of nonrice *Huangjiu*.

#### 3.4.3. OPLS-DA of *Huangjiu* from Different Regions

**Figure 6:**
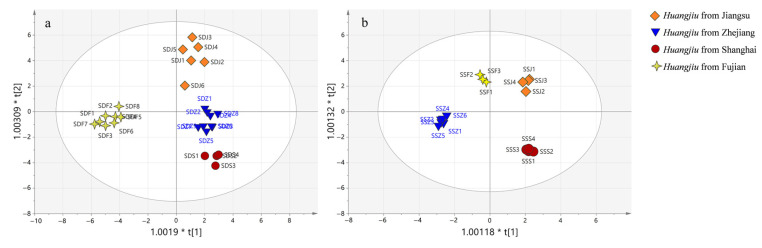
OPLS-DA of different regions of rice *Huangjiu,* (**a**) semidry *Huangjiu,* and (**b**) semisweet *Huangjiu*.

Different rice *Huangjiu* from different regions were distinguished by OPLS-DA (Figure 6). In semidry rice *Huangjiu* (Figure 6a R^2^Y = 0.936, Q^2^ = 0.752), a total of 22 carbonyl compounds with VIP > 1 and *p* < 0.05 were detected (Table 6). As shown in Figure 6a, the *Huangjiu* from the Fujian region was located in the negative half-axis, while the *Huangjiu* from the Zhejiang, Jiangsu, and Shanghai regions was located in the positive half-axis, indicating that the characteristics of carbonyl compounds in *Huangjiu* from the Fujian region were significantly different from those from other regions This might be due to the fact that the Fujian region used *Hongqu* as the saccharifying fermenter, while the other regions used wheat *Qu*, and the difference in microorganisms may lead to the difference in carbonyl compounds. As shown in Figure S4A, highly significant positive correlations existed between methyl glyoxal, (E)-2-butenal, and phenyl acetaldehyde, and semidry *Huangjiu* from the Fujian, Shanghai, and Zhejiang regions, respectively, whereas significant positive correlations existed between methional and semidry *Huangjiu* from the Jiangsu region.

Semisweet rice *Huangjiu* (Figure 6b; R^2^Y = 0.99, Q^2^ = 0.587) contained eight substances with VIP > 1 and *p* < 0.05 (Table 7). As shown in Figure S4B, highly significant positive correlations existed between phenyl acetaldehyde and semisweet *Huangjiu* from the Fujian region, as well as between 2-methylpropanal, 2-methylbutanal, 3-methylbutanal, and semisweet *Huangjiu* from the Jiangsu region. The compounds 2-pentanone and 4-heptanone were correlated with semisweet *Huangjiu* from the Shanghai region, whereas no carbonyl compounds correlating to semisweet *Huangjiu* from the Zhejiang region were discovered.

## 4. Conclusions

In the present study, a method for identifying carbonyl compounds in *Huangjiu* was developed by combining HS-SPME-GC-MS and PFBHA derivatization, and was applied to the targeted analysis of volatile carbonyl compounds in *Huangjiu*. The contents of most of the differential carbonyl compounds, such as benzaldehyde, 2-phenyl-2-butenal, 3-methylbutanal, 2-methylbutanal, and 2-furfural, were higher in semidry *Huangjiu* than in semisweet *Huangjiu*. The content of Strecker aldehydes (3-methylbutanal, benzaldehyde, 2-methylpropanal, and 2-methylbutanal) was higher in rice *Huangjiu* than in nonrice *Huangjiu*. Methyl glyoxal, (E)-2-butenal, and phenyl acetaldehyde could be used as representative carbonyl compounds in semidry rice *Huangjiu* from the Fujian, Shanghai, Zhejiang, and Jiangsu regions. In semisweet *Huangjiu*, the representative carbonyl compounds were phenylglyoxal from the Fujian region, 2-pentanone and 4-heptanone from the Shanghai region, and 2-methylpropanal, 2-methylbutanal, and 3-methylbutanal from the Jiangsu region. No representative carbonyl compounds were found in *Huangjiu* from the Zhejiang region.

## Figures and Tables

**Table 1:** Factor levels and experimental domain applied to optimize the HS-SPME experimental conditions.

Factor	Experimental Domain				
	−α ^a^	−1	0	+1	+α ^a^
PFBHA (g/L)	0.5	1	1.5	2	2.5
Extraction temperature (T_ex_-°C)	30	40	50	60	70
Extraction time (t_ex_-min)	20	30	40	50	60
Incubation time (t_inc_-min)	3	6	9	12	15

^a ^α = 2.000.

**Table 2:** Compounds identified in *Huangjiu*: retention time (RT), retention index (RI), *m*/*z* quantitative ion, identification method (ID).

RT (min)	RI_calc_^a^	RI_lit_^b^	Compounds	Ions (*m*/*z*)	ID
Alkanals					
15.53/15.69	1447/1453	1451/1457	Ethanal	181/209/239	STD/MS
16.87/17.10	1495/1504	1502/1509	Propanal	181/236/253	STD/MS
18.70/18.75	1558/1560	1572/1585	Butanal	181/239	STD/MS
21.01/21.28	1655/1666	1664/1675	Pentanal	181/239	STD/MS
22.90/23.08	1745/1754	1755/1765	Hexanal	181/239	STD/MS
24.89/25.06	1827/1833	1852/1861	Heptanal	181/239	STD/MS
26.89/27.03	1928/1935	1943/1950	Octanal	181/239	STD/MS
28.85/28.95	2027/2038	2041/2047	Nonanal	181/239	STD/MS
30.72/30.80	2127/2131	2138/2143	Decanal	181/239	STD/MS
32.55/32.62	2227/2231	2239/2243	Undecanal	181/239	STD/MS
34.30/34.35	2327/2329	2337/2340	Dodecanal	181/239	STD/MS
35.99	2426	2443	Tridecanal	181/239	STD/MS
Alkenals					
18.37/19.20	1544/1578	1566/1594	2-Propenal	181/251	STD/MS
22.19/23.20	1713/1759	1728/1763	(E)-2-Butenal	181/250	STD/MS
24.14/24.49	1801/1813	1808/1825	(E)-2-Pentenal	181/250	STD/MS
26.04/26.27	1886/1897	1898/1910	(E)-2-Hexenal	181/250	STD/MS
28.14/28.26	1987/1995	2009/2016	(E)-2-Heptenal	181/250	STD/MS
30.09	2093	2109	(E)-2-Octenal	181/250	STD/MS
31.92	2192	-	(E)-2-Nonenal	181/250	STD/MS
33.65/33.74	2290/2294	2307/2309	(E)-2-Decenal	181/250	STD/MS
Strecker aldehydes					
16.82/16.89	1493/1496	–	2-Methylpropanal	181/250	STD/MS
18.77/18.92	1561/1567	–	2-Methylbutanal	181/239	STD/MS
19.60/20.00	1594/1611	1599/1618	3-Methylbutanal	181/239	STD/MS
24.85/25.69	1928/1969	–	3-Methyl-2-butenal	181/264	STD/MS
30.77/30.83	2129/2133	2147/2150	Methional	181/299	STD/MS
33.20/33.26	2264/2267	–	Benzaldehyde	181/301	STD/MS
33.90/33.99	2303/2309	2326/2329	Phenyl acetaldehyde	181/91	STD/MS
Dicarbonyl					
32.97/34.26/35.67	2251/2324/2407	–	Diacetyl	181/279	STD/MS
35.74/36.17/36.32	2411/2437/2447	–	Methyl glyoxal	181/265	STD/MS
36.69/36.88	2469/2480	–	Glyoxal	181/251	STD/MS
Ketones					
15.98	1463	1466	Acetone	181/253	MS
17.20/17.26	1508/1511	1521/1521	2-Butanone	181/253	MS
17.55/18.04	1511/1531	–	3-Methyl-2-butanone	181/253	STD/MS
17.54/18.90	1511/1566	–	2-Pentanone	181/253	STD/MS
18.1192	1534	–	3-Pentanone	181/253	STD/MS
20.90/21.11	1650/1659	–	2-Hexanone	181/253	STD/MS
20.94	1651	–	4-Heptanone	181/253	STD/MS
22.46/22.73	1725/1738	–	2-Heptanone	181/253	STD/MS
24.33/24.67	1803/1819	1818/1833	2-Octanone	181/253	STD/MS
26.25/26.60	1895/1913	1925/1943	2-Nonanone	181/253	STD/MS
28.14/28.52	1986/2010	–	2-Decanone	181/253	STD/MS
Furans					
29.22/30.52	2047/2116	2056/2126	2-Furfural	181/291	STD/MS
30.64/31.65	2122/2177	–	5-Methyl-2-furfural	181/305	STD/MS
46.81/48.18	3158/3266	–	5-(Hydroxymethyl)furfural	181/321	STD/MS
Others					
23.12	1755	–	3-Penten-2-one	181/264	STD/MS
24.55/25.39	1813/1854	1838/1880	1-Octen-3- one	181/140	STD/MS
24.83	1825	–	Cyclopentanone	181/279	STD/MS
25.86	1862	–	Cyclohexanone	181/293	STD/MS
25.26/25.67	1841/1855	1864/1883	6-Methyl-5-hepten-2-one	181/253	STD/MS
28.36	1803	–	2-Cyclohexen-1-one	181/291	STD/MS
29.49/30.50	2061/2115	2092/2146	Acetoin	181/240	STD/MS
33.50/31.32	2159/2281	2171/2295	Acetophenone	181/315	STD/MS
35.03	2369	–	2-Phenyl-2-butenal	181/341	STD/MS
Internal standard					
33.33/33.39	2271/2274	–	*p*-Fluorobenzaldehyde	181/319	STD/MS

^a^ RI_calc_: retention indices calculated from C7 to C30 n-linear alkanes with VF-WAXms capillary column; ^b^ RI_lit_: retention indices reported in the literature for VF-WAXms capillary column or equivalent.–: retention indices reported in no-literature.

**Table 3:** Linear range, determination coefficient, limit of detection (LOD), and limit of quantitation (LOQ) of the proposed method.

Compounds	Linear Range (μg/L)	Determination Coefficient (r^2^)	LOD (μg/L)	LOQ (μg/L)
Propanal	0.785–785	0.9995	0.017	0.055
Butanal	0.074–74.2	0.9993	0.004	0.011
Pentanal	0.084–83.9	0.9999	0.016	0.059
Hexanal	0.081–81.1	0.9999	0.026	0.079
Heptanal	0.079–79.2	0.9999	0.011	0.038
Octanal	0.083–83.1	0.9999	0.006	0.023
Nonanal	0.242–121	0.9993	0.010	0.033
Decanal	0.026–26.4	0.9976	0.007	0.025
Undecanal	0.046–23.1	0.9997	0.004	0.015
Dodecanal	0.034–33.7	0.9980	0.007	0.026
Tridecanal	0.078–77.5	0.9994	0.006	0.021
2-Propenal	0.112–122	0.9999	0.029	0.098
(E)-2-Butenal	0.837–4185	0.9963	0.017	0.058
(E)-2-Pentenal	0.421–84.1	0.9997	0.021	0.065
(E)-2-Hexenal	0.082–81.8	0.9996	0.019	0.065
(E)-2-Heptenal	0.083–83.3	0.9998	0.024	0.075
(E)-2-Octenal	0.412–41.2	0.9992	0.061	0.194
(E)-2-Nonenal	0.419–41.9	0.9974	0.072	0.219
(E)-2-Decenal	0.176–44.0	0.9989	0.008	0.022
2-Methylpropanal	1.170–5825	0.9999	0.016	0.052
2-Methylbutanal	0.754–3770	0.9999	0.008	0.025
3-Methylbutanal	1.380–13,800	0.9999	0.043	0.135
3-Methyl-2-butenal	0.856–85.6	0.9997	0.008	0.035
Methional	0.500–500	0.9999	0.068	0.206
Benzaldehyde	2.350–11,750	0.9990	0.082	0.248
Phenyl acetaldehyde	0.923–46.2	0.9994	0.012	0.038
Diacetyl	1.042–1042	0.9994	0.085	0.265
Methyl glyoxal	0.450–2248	0.9994	0.022	0.068
Glyoxal	0.511–2556	0.9993	0.044	0.134
3-Methyl-2-butanone	0.567–56.7	0.9990	0.012	0.038
2-Pentanone	0.078–78.4	0.9996	0.012	0.029
3-Pentanone	0.312–156	0.9995	0.017	0.05
2-Hexanone	0.369–73.8	0.9999	0.002	0.007
4-Heptanone	0.059–59.0	0.9992	0.012	0.029
2-Heptanone	0.377–75.5	0.9999	0.062	0.187
2-Octanone	0.405–81.10	0.9999	0.022	0.066
2-Nonanone	0.170–85.0	0.9977	0.019	0.059
2-Decanone	0.152–76.0	0.9983	0.012	0.035
2-Furfural	4.647–46,470	0.9992	0.066	0.201
5-Methyl-2-furfural	0.113–5635	0.9993	0.012	0.046
3-Penten -2-one	1.608–8040	0.9998	0.080	0.243
1-Octen-3- one	0.042–84.0	0.9999	0.003	0.014
6-Methyl-5-hepten-2-one	0.069–69.0	0.9998	0.002	0.007
Cyclopentanone	0.084–4185	0.9962	0.004	0.017
Cyclohexanone	0.091–91.4	0.9990	0.009	0.029
2-Cyclohexen-1-one	0.448–89.6	0.9987	0.047	0.143
Acetoin	41.760–208,800	0.9988	0.053	0.162
Acetophenone	0.103–103	0.9992	0.011	0.027
2-Phenyl-2-butenal	0.750–3750	0.9998	0.105	0.317

**Table 4:** List of volatile carbonyl compounds significantly altered in semidry *Huangjiu* compared to semisweet *Huangjiu* (only signals *p* < 0.05 and VIP > 1 are presented).

Compound	VIP ^a^	*p*-Value ^b^	Semidry vs. Semisweet ^c^
2-Octanone	1.8062	<0.0001 (****)	⬆
(E)-2-Pentenal	1.7468	<0.0001 (****)	⬆
2-Hexanone	1.6892	<0.0001 (****)	⬆
2-Propenal	1.6827	<0.0001 (****)	⬇
2-Heptanone	1.5018	<0.0001 (****)	⬆
3-Pentanone	1.4325	0.0003 (***)	⬆
2-Phenyl-2-butenal	1.3881	0.0005 (***)	⬆
(E)-2-Nonenal	1.3733	0.0008 (***)	⬆
(E)-2-Heptenal	1.3592	<0.0001 (****)	⬆
Benzaldehyde	1.2966	0.0013 (**)	⬆
2-Furfural	1.2883	0.0005 (***)	⬆
3-Methyl-2-butanone	1.2679	0.0003 (***)	⬆
2-Methylbutanal	1.2543	0.0011 (**)	⬆
2-Cyclohexen-1-one	1.2394	0.0004 (***)	⬆
3-Methylbutanal	1.1973	0.0033 (**)	⬆
Dodecanal	1.1845	0.0037 (**)	⬆
4-Heptanone	1.1236	0.0061 (**)	⬆
Decanal	1.1213	0.0062 (**)	⬇
Glyoxal	1.0612	0.0100 (**)	⬇

^a^ VIP: variable importance in the projection; ^b^ *p*-value: significances between date; ** p < 0.01, *** p < 0.001, **** p < 0.0001; ^c^ “⬆/⬇” means increase/decrease of the compounds.

**Table 5:** List of volatile carbonyl compounds significantly altered in rice *Huangjiu* compared to nonrice *Huangjiu* (only signals *p* < 0.05 and VIP > 1 are presented).

Compound	VIP ^a^	*p*-Value ^b^	Rice vs. Nonrice ^c^
(E)-2-Hexenal	1.8955	0.0002 (***)	⬇
3-Methylbutanal	1.7378	<0.0001 (****)	⬆
Benzaldehyde	1.6881	<0.0001 (****)	⬆
2-Nonanone	1.6221	<0.0001 (****)	⬆
Cyclopentanone	1.5839	0.0041 (**)	⬇
2-Decanone	1.5299	0.0066 (**)	⬆
2-Methylpropanal	1.5233	<0.0001 (****)	⬆
(E)-2-Heptenal	1.5024	0.0077 (**)	⬇
2-Methylbutanal	1.4562	0.0001 (***)	⬆
3-Methyl-2-butanone	1.3595	0.0004 (***)	⬆
(E)-2-Butenal	1.2837	0.0008 (***)	⬆
2-Butanone	1.2357	0.0013 (**)	⬆
Butanal	1.2134	0.0056 (**)	⬆
Methyl glyoxal	1.1814	0.0072 (**)	⬇
2-Hexanone	1.0771	0.0157 (*)	⬆
2-Phenyl-2-butenal	1.0529	0.0083 (**)	⬆
2-Propenal	1.0508	0.0187 (*)	⬇
2-Heptanone	1.0428	0.0198 (*)	⬆
(E)-2-Octenal	1.0178	0.0232 (*)	⬇
Diacetyl	1.0071	0.0249 (*)	⬇

^a^ VIP: variable importance in the projection; ^b^ *p*-value: significances between date; * *p* < 0.05, ** p < 0.01, *** p < 0.001, **** p < 0.0001; ^c^ “⬆/⬇” means increase/decrease of the compounds.

**Table 6:** OPLS-DA results showing the critical volatile carbonyl compounds (variable importance in projection VIP > 1.0, *p* < 0.05) present across semidry *Huangjiu* samples of different regions (Zhejiang, Jiangsu, Shanghai, Fujian).

Compound	VIP ^a^	*p*-Value ^b^
2-Phenyl-2-butenal	1.7335	<0.0001 (****)
Cyclopentanone	1.5191	<0.0001 (****)
Glyoxal	1.3979	0.0003 (***)
2-Decanone	1.3123	<0.0001 (****)
Benzaldehyde	1.3065	0.0006 (***)
2-Cyclohexene-1-one	1.2443	<0.0001 (****)
(E)-2-Butenal	1.2438	<0.0001 (****)
Dodecanal	1.2284	0.0023 (**)
Heptanal	1.2206	<0.0001 (****)
Decanal	1.2165	0.0048 (**)
Pentanal	1.1786	<0.0001 (****)
Methional	1.1758	0.0023 (**)
Undecanal	1.1545	0.0048 (**)
2-Pentanone	1.1265	0.046 (*)
6-Methyl-5-hepten-2-one	1.1235	0.0094 (**)
3-Methyl-2-butenal	1.121	0.0069 (**)
Methyl glyoxal	1.1079	<0.0001 (****)
3-Methyl-2-butanone	1.0926	0.001 (***)
Phenyl acetaldehyde	1.0685	<0.0001 (****)
2-Heptanone	1.0438	0.0023 (**)
Propanal	1.0069	0.0004 (***)
Hexanal	1.0064	0.0033 (**)

^a^ VIP: variable importance in the projection; ^b^ *p*-value: significances between date; * *p* < 0.05, ** *p* < 0.01, *** *p* < 0.001, **** *p* < 0.0001.

**Table 7:** OPLS-DA results showing the critical volatile carbonyl compounds (variable importance in projection VIP > 1.0, *p* < 0.05) present across semisweet *Huangjiu* samples of different regions (Zhejiang, Jiangsu, Shanghai, Fujian).

Compound	VIP ^a^	*p*-Value ^b^
Phenyl acetaldehyde	1.5676	0.0002 (***)
2-Pentanone	1.5134	0.0009 (***)
2-Methylbutanal	1.4287	0.0064 (**)
2-Octanone	1.3833	0.0445 (*)
2-Methylpropanal	1.3766	0.0102 (*)
4-Heptanone	1.2948	0.0178 (*)
3-Methyl-2-butenal	1.2901	0.0375 (*)
3-Methylbutanal	1.2514	0.0375 (*)

^a^ VIP: variable importance in the projection; ^b^ *p*-value: significances between date; * *p* < 0.05, ** p < 0.01, *** p < 0.001.

## Data Availability

The data are available from the corresponding author.

## Supplementary Materials

The following supporting information can be downloaded at: https://www.mdpi.com/article/10.3390/foods12071455/s1, Figure S1: Effect of sample volume on extraction efficiency; Figure S2: Effect of ionic strength on extraction efficiency; Figure S3: The result of the corresponding optimizer; Figure S4: The OPLS-DA biplot of *Huangjiu* from different regions; Table S1: Experimental conditions and response values (total area) of the CCD used to optimize the derivatization conditions of volatile compounds in *Huangjiu*; Table S2: ANOVA study for responses; Table S3: Precision and accuracy of the proposed method; Table S4: The range of volatile carbonyl compounds in semi-dry *Huangjiu* from different production areas; Table S5: The range of volatile carbonyl compounds in semi-sweet *Huangjiu* from different production areas.
