# Supplementary material for: Differentiating Huangjiu with Varying Sugar Contents from Different Regions Based on Targeted Metabolomics Analyses of Volatile Carbonyl Compounds
Source: Foods. 2023 Mar 29;12(7):1455. doi: 10.3390/foods12071455 (PMC10094199; doi:10.3390/foods12071455)
Supplement: Supplementary file 1 [file foods-12-01455-s001.zip › Supplementary Figure.pdf]

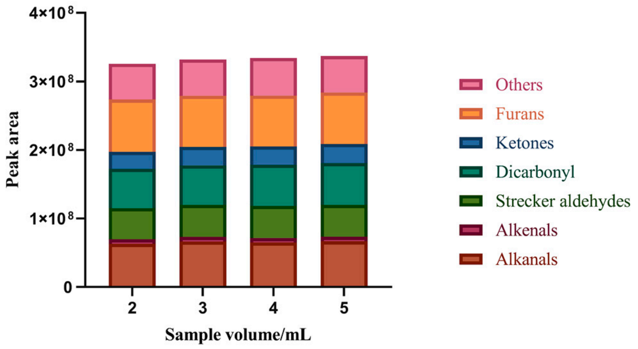

Figure S1. Effect of sample volume on extraction efficiency

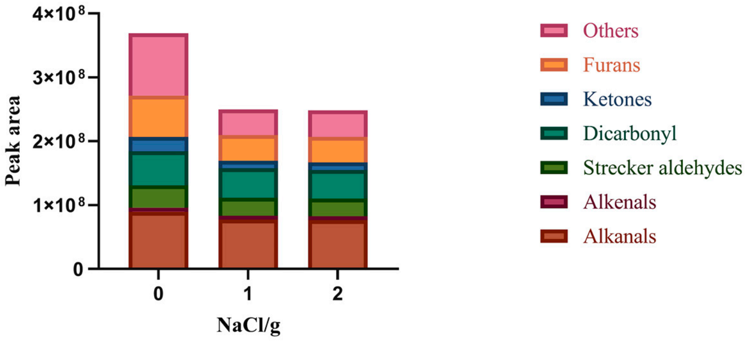

Figure S2. Effect of ionic strength on extraction efficiency

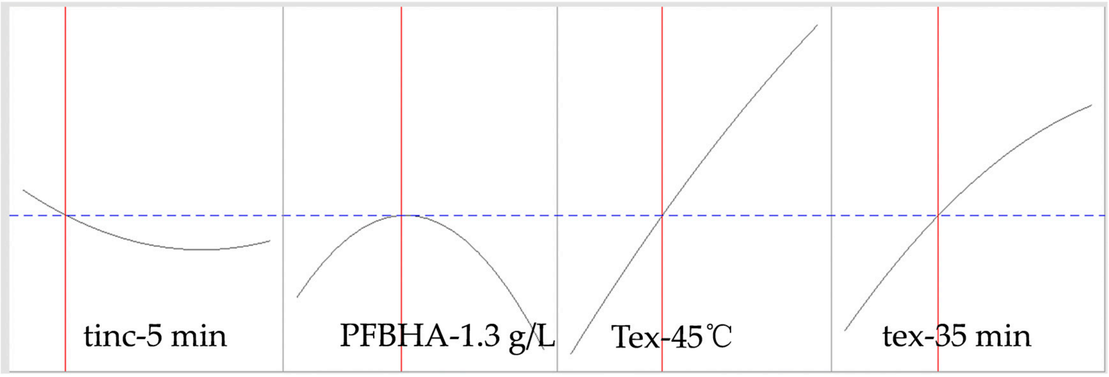

Figure S3. The result of the corresponding optimizer

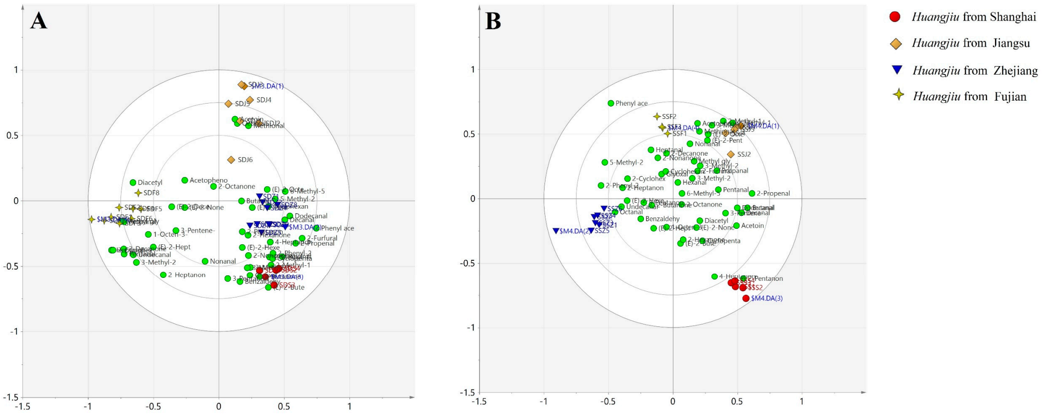

Figure S4. The OPLS-DA biplot of *Huangjiu* from different regions.
(A)Semi-dry *Huangjiu* from different regions, and (B) semi-sweet *Huangjiu* from different regions
