# Supplementary material for: Differentiating Huangjiu with Varying Sugar Contents from Different Regions Based on Targeted Metabolomics Analyses of Volatile Carbonyl Compounds
Source: Foods. 2023 Mar 29;12(7):1455. doi: 10.3390/foods12071455 (PMC10094199; doi:10.3390/foods12071455)
Supplement: Supplementary file 1 [file foods-12-01455-s001.zip › Supplementary Table.pdf]

Table S1. Experimental conditions and response values (total area) of the CCD used to optimize the derivatization conditions of volatile compounds in *Huangjiu*.

Run	t_{inc}（min）[a]	PFBHA (g/L)	T_{ex}(℃)[b]	t_{ex} (min)[c]	Response value (total area)[d]
1	6	1	40	30	261334010
2	12	1	40	30	254926762
3	6	2	40	30	255650273
4	12	2	40	30	223717408
5	6	1	60	30	341573948
6	12	1	60	30	328045601
7	6	2	60	30	369272000
8	12	2	60	30	334673316
9	6	1	40	50	359097201
10	12	1	40	50	333905880
11	6	2	40	50	263236801
12	12	2	40	50	248411763
13	6	1	60	50	486483188
14	12	1	60	50	434496667
15	6	2	60	50	434562786
16	12	2	60	50	397470405
17	3	1.5	50	40	393053751
18	15	1.5	50	40	350110046
19	9	0.5	50	40	290108195
20	9	2.5	50	40	262764688
21	9	1.5	30	40	203081000
22	9	1.5	70	40	474896338
23	9	1.5	50	20	256300448
24	9	1.5	50	60	401737386
25	9	1.5	50	40	367972080
26	9	1.5	50	40	354400686
27	9	1.5	50	40	342805869
28	9	1.5	50	40	358260426
29	9	1.5	50	40	346709151

[a]t_{inc}-incubation time.

[b]T_{ex}- extraction and incubation temperature.

[c]t_{ex}-extraction time.

[d]Total area in expressed in arbitrary units.

Table S2. ANOVA study for responses.

Source	Sum of squares	df	Mean square	F value	p-value Prob>F	Remarks
Model	1.50×10^{17}	14	1.07×10^{16}	72.81	< 0.0001	Significant
A-tinc	3.79×10^{15}	1	3.79×10^{15}	25.68	0.0002	
B-PFBHA	4.47×10^{15}	1	4.47×10^{15}	30.33	< 0.0001	
C-Tex	9.00×10^{16}	1	9.00×10^{16}	610.72	< 0.0001	
D-tex	3.22×10^{16}	1	3.22×10^{16}	218.56	< 0.0001	
AB	2.85×10^{13}	1	2.85×10^{13}	0.19	0.6671	
AC	2.16×10^{14}	1	2.16×10^{14}	1.47	0.2457	
AD	1.14×10^{14}	1	1.14×10^{14}	0.77	0.3949	
BC	1.67×10^{15}	1	1.67×10^{15}	11.35	0.0046	
BD	4.48×10^{15}	1	4.48×10^{15}	30.39	< 0.0001	
CD	1.82×10^{15}	1	1.82×10^{15}	12.31	0.0035	
A^2	6.60×10^{14}	1	6.60×10^{14}	4.47	0.0528	
B^2	9.12×10^{15}	1	9.12×10^{15}	61.84	< 0.0001	
C^2	2.50×10^{14}	1	2.50×10^{14}	1.70	0.2135	
D^2	8.13×10^{14}	1	8.13×10^{14}	5.52	0.0340	
Residual	2.06×10^{15}	14	1.47×10^{14}			
Lack of Fit	1.67×10^{15}	10	1.67×10^{14}	1.71	0.3199	not significant
Pure Error	3.92×10^{14}	4	9.80×10^{13}			
Cor Total	1.52×10^{17}	28		72.81		

Table S3. Precision and accuracy of the proposed method.

Compounds	Precision(%, n=6)		Accuracy(%,n=3)					
			Low		Mid		High	
	Intraday precision	Interday precision	Concentration（μg/L）	Recovery	Concentration（μg/L）	Recovery	Concentration（μg/L）	Recovery
Propanal	1.1	3.3	4.6	101.4±5.9	21.3	100.2±0.3	40.0	106.5±4.3
Butanal	4.3	7.5	0.2	106.8±6.3	6.7	108.0±6.1	20.8	99.9±2.1
Pentanal	6.0	6.4	0.9	98.4±6.9	25.5	98.0±5.2	79.2	91.3±9.1
Hexanal	6.4	8.4	3.2	100.6±9.4	10.9	102.9±7.1	27.6	107.2±5.0
Heptanal	4.2	3.5	0.4	99.6±5.7	10.5	93.0±2.9	32.7	106.3±10.4
Octanal	8.7	7.2	0.2	107.5±0.7	5.6	102.1±2.7	17.5	100.2±3.4
Nonanal	5.0	7.5	0.5	93.1±9.6	12.9	105.0±6.7	40.0	98.2±9.7
Decanal	5.7	6.8	0.5	98.4±3.2	14.6	97.7±7.7	45.4	95.5±4.0
Undecanal	3.9	7.3	0.8	96.8±1.4	23.5	111.3±3.5	73.0	100.3±5.4
Dodecanal	7.4	9.8	0.3	101.4±1.7	7.4	98.7±5.6	22.8	95.6±8.4
Tridecanal	7.5	9.1	0.3	97.2±5.0	7.1	102.4±5.8	22.0	97.8±3.3
2-Propenal	6.4	7.8	1.2	100.5±3.2	34.7	103.2±7.7	108.0	97.9±2.5
(E)-2-Butenal	2.2	4.5	17.6	100.7±1.2	60.7	105.6±3.2	154.0	97.7±0.8
(E)-2-Pentenal	4.7	6.6	1.4	105.8±2.3	37.9	97.6±5.3	117.0	96.9±1.6
(E)-2-Hexenal	4.9	7.0	0.3	94.7±5.3	7.5	101.3±7.0	23.1	108.6±2.0
(E)-2-Heptenal	7.6	8.5	0.2	107.9±3.5	5.6	97.9±7.4	17.5	94.1±11.9
(E)-2-Octenal	3.4	7.3	0.3	94.5±7.1	7.3	102.0±2.3	22.5	101.0±7.0
(E)-2-Nonenal	3.3	7.1	0.3	100.3±5.8	8.00	99.1±4.1	24.8	93.4±8.4
(E)-2-Decenal	5.1	8.8	0.3	98.4±7.5	7.2	92.8±4.5	22.3	110.2±4.0
2-Methylpropanal	6.5	8.1	10.6	100.0±4.9	149.0	99.4±4.8	924.0	103.6±5.0
2-Methylbutanal	4.5	6.9	7.1	99.6±1.9	100.0	100.6±5.5	620.0	100.4±2.8

3-Methylbutanal	9.3	7.9	25.4	101.1±4.6	357.0	100.0±6.2	2214.0	96.6±4.2
3-Methyl-2-butenal	6.0	7.5	0.2	103.1±5.6	5.9	95.9±5.4	18.3	102.9±1.7
Methional	3.4	3.7	8.8	100.3±3.3	30.4	105.1±3.2	77.2	99.2±2.3
Benzaldehyde	5.3	3.9	32.1	105.9±2.5	453.0	102.8±5.7	2806.0	103.2±2.0
Phenyl acetaldehyde	4.3	9.4	17.1	94.7±4.8	58.9	100.1±4.3	149.0	100.1±2.8
Diacetyl	5.6	8.5	3.4	101.1±11.0	11.8	100.6±6.7	29.9	97±1.6.0
Methyl glyoxal	2.4	2.6	4.0	99.9±3.8	13.7	101.2±2.1	34.7	94.8±2.5
Glyoxal	6.0	6.4	2.5	102.3±6.7	34.9	94.5±1.9	216.0	100.7±7.4
3-Methyl-2-butanone	3.1	5.7	0.5	106.4±8.1	14.9	98.0±0.8	46.2	104.3±8.5
2-Pentanone	7.7	8.1	0.3	96.1±5.8	7.1	102.1±4.0	22.0	97.1±6.1
3-Pentanone	5.2	8.3	0.5	100.3±10.5	15.1	106.0±5.7	46.8	98.8±8.8
2-Hexanone	6.2	6.6	0.2	95.5±6.9	5.0	95.8±8.1	15.5	96.6±4.9
4-Heptanone	5.8	9.3	0.4	104.0±3.3	12.5	108.8±6.9	38.6	101.8±10.0
2-Heptanone	8.1	9.6	0.4	95.4±4.3	12.2	99.6±8.8	37.7	98.6±6.7
2-Octanone	5.4	6.4	0.4	99.9±1.9	11.4	96.4±5.2	35.5	100.2±10.7
2-Nonanone	5.2	5.5	0.3	103±1.4	8.9	99.3±4.0	27.6	98.1±1.6
2-Decanone	7.6	9.3	0.3	102.9±6.4	7.4	98.7±5.6	22.8	91.2±1.9
2-Furfural	6.2	7.3	65.9	96.5±2.5	928.0	100.6±2.8	5752.0	99.9±2.2
5-Methyl-2-furfural	6.4	4.9	9.0	99.5±7.0	30.9	106.4±2.9	78.3	97.7±2.3
3-Penten-2-one	4.0	4.6	7.9	98.9±4.2	111.0	99.4±2.6	687.0	100.0±4.0
1-Octen-3- one	7.3	6.6	0.3	91.2±3.2	7.6	93.8±8.3	23.7	99.1±10.4
6-Methyl-5-hepten-2-one	5.2	4.5	0.5	97.6±5.1	13.8	100.1±4.5	42.8	96.0±3.1
Cyclopentanone	3.9	8.5	0.3	97.0±1.0	7.1	108.6±3.2	22.0	98.7±1.4
Cyclohexanone	7.4	9.8	0.3	101.4±5.5	7.6	96.3±5.4	23.4	105.8±4.1
2-Cyclohexen-1-one	4.4	7.6	0.3	100.0±2.0	7.9	102.5±5.6	24.5	98.3±8.0
Acetoin	3.5	5.9	101.0	102.3±1.7	7114.0	96.4±2.3	15650.0	99.1±0.5

Acetophenone	3.5	3.8	1.3	101.2±7.4	35.7	98.0±1.9	111.0	106.0±1.8
2-Phenyl-2-butenal	6.3	7.0	3.9	102.9±3.8	54.9	101.0±4.3	340.0	98.7±4.5

Table S4. The range of volatile carbonyl compounds in semi-dry *Huangjiu* from different production areas.

Compound（μg/L）	SDJ (n=6)		SDZ (n=9)		SDS (n=4)		SDF (n=8)	
	Concentration range	Mean±SD	Concentrati on range	Mean±SD	Concentrati on range	Mean±SD	Concentrati on range	Mean±SD
Propanal	30.35~61	44.5^{b}±10.49	38.03~89.44	58.4^{b}±15.17	38.16~64.43	51.27^{b}±11.22	35.31~336.92	179^{a}±103.3
Butanal	3.34~16.84	9.45^{a}±5.12	3.17~23.74	8.96^{a}±6.15	5.62~17.46	9.77^{a}±5.34	0.33~12.58	7.51^{a}±4.14
Pentanal	8.82~26.65	18.07^{c}±7.04	10.67~37.58	25.66^{bc}±9.18	33.38~40.65	37.02^{b}±3.86	35.55~84.82	57.13^{a}±16.76
Hexanal	18.9~45.23	30.33^{b}±10.43	21.73~70.92	47.49^{a}±13.78	43.58~55.68	49.18^{a}±5.10	21.67~42.98	30.74^{b}±7.85
Heptanal	0.54~1.1	0.83^{b}±0.3	0.89~1.85	1.29^{b}±0.30	1.16~1.73	1.42^{b}±0.26	1.78~4.01	2.8^{a}±0.75
Octanal	0.4~6.74	2.53^{a}±3.18	0.35~0.56	0.46^{ab}±0.08	0.34~0.51	0.43^{b}±0.08	0.17~0.57	0.37^{b}±0.13
Nonanal	0.26~4.11	1.43^{a}±1.43	nq~6.9	3.7^{a}±2.20	1.41~9.43	4.17^{a}±3.76	nq~8.75	3.94^{a}±2.88
Decanal	2.29~8.28	4.84^{ab}±3	4.15~13.77	8.42^{a}±3.42	4.1~6.36	5.27^{ab}±0.94	0.63~8.49	3.35^{b}±2.45
Undecanal	nd~0.53	0.11^{b}±0.22	1.27~10.67	4.18^{b}±3.34	nd~6.33	3.31^{b}±2.7	3.2~14.34	9.15^{a}±3.65
Dodecanal	4.52~11.51	7.3^{b}±2.57	1.99~10.7	6.23^{b}±3.13	8.94~16.27	11.5^{a}±3.30	1.64~9.16	4^{b}±2.4
Tridecanal	nd	nd^{a}	nd	nd^{a}	nd	nd^{a}	nd	nd^{a}
2-Propenal	0.83~2.12	1.43^{ab}±0.49	0.38~5.49	2.94^{a}±1.9	1.87~3.76	2.95^{a}±0.82	0.21~1.42	0.83^{b}±0.47
(E)-2-Butenal	88.76~241.7	140.4^{c}±54.6	172.7~403.6	294.3^{b}±83.58	218.7~663.6	498.6^{a}±248.5	104.6~281.1	206.3^{bc}±58.32
(E)-2-Pentenal	3.24~4.92	3.75^{a}±0.63	2.38~5.5	3.89^{a}±0.95	2.28~4.93	3.77^{a}±1.15	1.26~4.41	3.28^{a}±1.10
(E)-2-Hexenal	0.31~2.93	1.03^{a}±0.98	0.68~5.31	2.24^{a}±1.43	1.24~3.54	2.1^{a}±1.06	0.62~2.93	1.65^{a}±0.75
(E)-2-Heptenal	0.1~0.14	0.11^{b}±0.02	0.11~0.16	0.13^{ab}±0.03	0.1~0.17	0.13^{ab}±0.04	0.13~0.22	0.16^{a}±0.04
(E)-2-Octenal	0.29~0.38	0.33^{a}±0.05	0.29~0.35	0.32^{a}±0.03	0.3~0.36	0.34^{a}±0.04	0.3~0.33	0.31^{a}±0.02
(E)-2-Nonenal	0.37~0.47	0.42^{a}±0.05	0.38~0.53	0.45^{a}±0.06	0.38~0.44	0.41^{a}±0.04	0.39~0.65	0.47^{a}±0.2
(E)-2-Decenal	nd	nd^{a}	nd	nd^{a}	nd	nd^{a}	nd~1.38	0.22^{a}±0.48
2-Methylpropanal	687.3~1273	913.1^{b}±230.8	895.8~1606	1151^{ab}±251.6	1008~2413	1647 ^{a}±585.4	379.5~1752	844.5^{b}±483.5
2-Methylbutanal	558.9~968.7	727.7^{b}±153.5	734.6~1437	1027^{ab}±261.6	1100~1721	1365^{a}±259.9	348.7~1501	757.7^{b}±382.5
3-Methylbutanal	2183~3777	2868^{b}±651	2792~5408	4087^{ab}±916.5	4588~6560	5405^{a}±875.6	1393~6033	3469^{b}±1794

3-Methyl-2-butenal	2.89~7.15	4.29^{b}±1.94	3.48~5.72	5.04^{b}±0.80	5.33~9.72	7.94^{a}±1.87	2.91~6.82	4.89^{b}±1.66
Methional	nq~733.6	435.75^{a}±304.5	61~211.2	124.7^{b}±53.26	120.2~250.2	169^{b}±59.1	nd~241.6	97.14^{b}±90.03
Benzaldehyde	743.5~1934	129^{b}±465.6	2295~4893	3610^{a}±932.6	2471~4048	3226^{a}±712.6	1569~4911	2555^{ab}±1135
Phenyl acetaldehyde	122.7~216.4	167.5^{ab}±37.58	152.6~269.6	214.9^{a}±39.29	214.1~230.5	220.8^{a}±7.14	55.22~196.1	110.7^{b}±51.52
Diacetyl	10.63~199.6	122^{ab}±80.82	14.82~88.55	47.7^{b}±22.97	32.06~63.35	48.52^{b}±12.90	3.38~318.8	201.2^{a}±109.5
Glyoxal	163.8~1164	670.4^{b}±369.6	367.7~1377	784.7^{b}±353.7	1329~2372	1833^{a}±532	420.5~1342	767.1^{b}±309.1
Methyl glyoxal	8.51~858.4	327.8^{b}±291.2	61.91~394.8	213.3^{b}±120.8	391.1~637.2	499.9^{b}±105.8	326.5~2231	1174^{a}±587.8
2-Butanone*	270~682.4	468.9^{c}±180.3	41.71~3198	1462 ^{ab}±982.5	1145~2287	1811^{a}±482	593.1~1397	882.1^{bc}±301.5
3-Methyl-2-butanone	2.4~9.91	6.12^{c}±2.85	5.05~27.3	14^{bc}±7.44	14.67~30.03	20.79^{ab}±6.85	16.88~54.38	31.74^{a}±12.99
2-Pentanone	3.17~8.24	5.23^{b}±1.8	2.01~7.21	4.72^{b}±1.85	4.52~12.15	8.18^{a}±3.95	4.29~5.76	4.96^{b}±0.55
3-Pentanone	0.87~9.3	4.74^{b}±3.6	4.86~21.97	10.47^{ab}±5.28	10.67~15.55	14.3^{ab}±2.43	3.03~15.48	9.87^{ab}±4.35
2-Hexanone	1.19~4.71	2.65^{a}±1.33	0.11~4.91	3.44^{a}±1.39	3.09~5.3	3.97^{a}±0.96	1.45~4.54	2.83^{a}±1.24
4-Heptanone	1.49~8.81	4.19^{a}±3.25	3.17~14.17	7.61^{a}±3.63	4.69~12.09	7.47^{a}±3.26	2.01~6.54	4.14^{a}±1.62
2-Heptanone	2.85~6.26	4.57^{b}±1.29	5.87~22.29	10.49^{a}±5.25	10.8~11.55	11.22^{a}±0.33	7.25~20.6	14.32^{a}±4.67
2-Octanone	0.67~1.82	1.3^{a}±0.42	0.53~1.38	1.13^{a}±0.28	0.9~1.54	1.25^{a}±0.30	0.66~1.87	1.26^{a}±0.45
2-Nonanone	1.13~13.97	7.53^{b}±4.67	2.23~18.18	9.26^{ab}±4.70	9.17~26.36	15.61^{a}±7.58	5.41~10.93	8.27^{b}±2.24
2-Decanone	nd	nd^{a}	nd	nd^{a}	0.21~0.24	0.22^{a}±0.02	0.22~0.77	0.38^{a}±0.3
2-Furfural	9267~26501	17916^{bc}±7348	9743~37037	24202^{ab}±8346	25548~29652	27121^{a}±1934	8995 ~16934	12419^{c}±2652
5-Methyl-2-furfural	68.08~154.7	124^{ab}±31.56	117.8~171.9	145.1^{a}±20.99	101.5~150.1	119.28^{ab}±21.36	47.19~160.8	100.2^{b}±40.72
3-Pentene-2-one	565.9~1563	1069^{a}±362.2	651.~1625.6	1111^{a}±377	920.7~1622	1267^{a}±287	933.6~1784	1368^{a}±299.4
1-Octen-3- one	0.07~0.08	0.07^{b}±1	0.07~0.18	0.09^{ab}±0.04	0.08~0.09	0.09^{ab}±0.02	0.09~0.21	0.13^{a}±0.06
6-Methyl-5-hepten-2-one	0.19~1.09	0.63^{a}±0.37	0.3~0.86	0.49^{b}±0.3	0.7~0.91	0.8^{a}±0.10	0.19~0.5	0.33^{b}±0.2
2-Cyclohexen-1-one	0.02~0.04	0.03^{c}±0.02	0.04~0.08	0.06^{bc}±0.02	0.09~0.14	0.11^{b}±0.03	0.14~0.36	0.24^{a}±0.09
Cyclopentanone	1.86~9.29	4.24^{b}±2.100	2.28~6.1	3.87^{b}±1.47	7.67~11.81	9.96^{a}±2.02	1.67~5.3	3.26^{b}±1.20
Cyclohexanone	5.32~8.04	6.27^{a}±2	5.49~8.05	6.87^{a}±0.82	6.44~6.96	6.65^{a}±0.26	2.52~9.26	5.45^{a}±2.37
Acetoin	56010~107267	72713^{a}±21387	14992~83110	41284^{b}±20640	19636~38068	27832^{b}±7637	5356~77376	36455^{b}±25054

Acetophenone	10.3~120.7	42.2[a]±31.0	16.9~49.6	29.8[a]±10.3	18.7~36.8	27.7[a]±20.0	13.7~93.0	45.7[a]±28.8
2-Phenyl-2-butenal	178.7~582.6	387.7[b]±172.6	722.7~1391	1100[a]±239.1	547.8~905.5	646[b]±173.6	232.5~728.8	515.8[b]±180.5

* area/area of internal standard.

nq: not quantified, nd: not detected.

Table S5. The range of volatile carbonyl compounds in semi-sweet *Huangjiu* from different production areas.

Compound（μg/L）	SSJ (n=4)		SSZ (n=6)		SSS (n=4)		SSF (n=3)		SSN (n=11)	
	Concentration range	Mean±SD	Concentration range	Mean±SD	Concentration range	Mean±SD	Concentration range	Mean±SD	Concentration range	Mean±SD
Propanal	40.33~80.58	58.46^{ab}±18.09	10.14~68.83	39.44^{b}±24.66	21.12~105.3	63.3^{ab}±34.62	66.01~94.68	81.07^{a}±14.39	11.19~79.55	45.86^{ab}±19.54
Butanal	3.78~12.9	7.83^{ab}±3.78	1.33~8.1	4.06^{bc}±2.54	6.87~12	9.16^{a}±2.56	2.37~10.93	6.59^{abc}±4.28	0.56~6.41	2.97^{c}±2.18
Pentanal	16.55~47.7	27.17^{a}±14.24	9.69~25.01	14.8^{a}±5.95	8.14~72.17	28.78^{a}±29.28	12.91~35.09	27.56^{a}±12.69	8.53~39.79	19.76^{a}±9.26
Hexanal	27.35~43.78	35.12^{a}±6.75	22.09~45.26	30.9^{a}±8.24	15.6~76.73	31.23^{a}±30.34	19.09~63.88	35.78^{a}±24.47	10.69~61.59	32.91^{a}±14.27
Heptanal	0.68~1.69	1.13^{a}±0.42	0.63~1.99	1.18^{a}±0.5	0.51~1.4	0.9^{a}±0.45	1.03~3.15	1.9^{a}±1.11	0.68~2.72	1.73^{a}±0.79
Octanal	0.43~0.6	0.51^{a}±0.07	0.42~5.49	1.84^{a}±2.18	0.27~0.51	0.39^{a}±0.11	0.43~0.52	0.49^{a}±0.05	0.27~1.93	0.74^{a}±0.54
Nonanal	1.76~16.57	7.19^{a}±6.86	0~6.74	3.92^{a}±3.14	0.56~7.14	4.03^{a}±3.01	7.32~12.14	10.25^{a}±2.57	0.6~17.21	6.51^{a}±5.71
Decanal	9.34~16.85	12.58^{ab}±3.16	2.59~10.39	6.43^{ab}±3.94	4.37~25.27	13.1^{a}±9.75	6.83~7.73	7.37^{ab}±0.48	0.08~9.09	5.41^{b}±2.89
Undecanal	0.73~4.66	2.25^{a}±1.78	0~9.27	4.58^{a}±3.86	0.91~3.01	2.19^{a}±0.94	1.29~5.23	2.79^{a}±2.13	0~5.85	1.59^{a}±2.08
Dodecanal	2.81~6.99	4.82^{a}±1.79	0.75~5.92	2.34^{a}±1.91	1.58~9.93	4.85^{a}±3.57	2.09~3.22	2.53^{a}±0.6	0~23.17	3.39^{a}±6.82
Tridecanal	nd	nd^{a}	nd	nd^{a}	nd	nd^{a}	nd	nd^{a}	nd	nd^{a}
2-Propenal	6.24~13.18	9.33^{a}±3	2.04~5.42	3.59^{a}±1.17	3.98~15.63	7.68^{a}±5.39	2.35~6.63	4.18^{a}±2.2	3.19~234.59	48.86^{a}±71.03
(E)-2-Butenal	224.1~620	380.8^{a}±174.7	130.6~744.3	384.8^{a}±234.9	312.8~483.4	400^{a}±90.61	76.45~128.9	97.94^{b}±27.5	69.5~272.1	137.3^{b}±68.13
(E)-2-Pentenal	2.26~3.52	2.97^{a}±0.65	1.44~2.46	2.06^{a}±0.45	1.15~2.53	1.95^{a}±0.67	1.59~2.99	2.19^{a}±0.72	0.3~5.92	1.25^{a}±1.68
(E)-2-Hexenal	0.5~1.31	0.87^{b}±0.34	0.55~2.7	1.38^{b}±0.75	0.69~1.62	1.05^{b}±0.41	1.18~1.71	1.36^{b}±0.3	0.73~9.86	5.07^{a}±2.32
(E)-2-Heptenal	0.1~0.11	0.11^{a}±0.01	0.1~0.12	0.11^{a}±0.01	0.1~0.11	0.11^{a}±0	0.1~0.1	0.1^{a}±0	0.13~0.48	0.21^{a}±0.11
(E)-2-Octenal	0.33~0.38	0.36^{a}±0.02	0.3~0.36	0.33^{a}±0.02	0.32~0.35	0.33^{a}±0.01	0.32~0.43	0.37^{a}±0.05	0.32~1.18	0.49^{a}±0.24
(E)-2-Nonenal	0.34~0.45	0.38^{a}±0.05	0.35~0.4	0.37^{a}±0.02	0.33~0.56	0.39^{a}±0.11	0.33~0.35	0.34^{a}±0.01	0.34~0.64	0.43^{a}±0.09
(E)-2-Decenal	nd	nd^{a}	nd	nd^{a}	nd	nd^{a}	nd	nd^{a}	nq~0.26	0.04^{a}±0.08
2-Methylpropanal	1016~1582	1281^{a}±235.2	233.1~950.4	548.7^{c}±271.3	315.4~1053	676.6^{bc}±318.5	631.3~1395	1019^{ab}±382.2	191.3~387.6	308.7^{c}±63.41
2-Methylbutanal	649.3~1188	941.1^{a}±231.5	254.1~746.1	455^{bc}±178.8	185.1~650.7	446.7^{bc}±194.3	568.7~778.5	643.3^{b}±117.3	166.3~476	268.6^{c}±89.44
3-Methylbutanal	3216~4631	3788^{a}±689.4	1155~3443	2065^{b}±850.8	789.8~3577	1921^{b}±1188	2310~3829	2840^{ab}±856.7	348.9~933.8	705.4^{c}±184.9
3-Methyl-2-butenal	6.11~7.39	6.95^{a}±0.58	3.2~8.1	5.1^{a}±1.81	3.63~5.42	4.62^{a}±0.75	2.57~5.82	4.07^{a}±1.64	1.88~9.42	4.43^{a}±2.54

Methional	311~1477	741^{ab}±536.4	83.62~336	169.7^{b}±102.2	195.5~272.7	247.5^{b}±35.57	195~2389	1041^{a}±1180	nd~628	213.3^{b}±183.4
Benzaldehyde	904~2450	1438^{a}±690.5	501.4~2917	1946^{a}±1089	126.2~2404	1427^{a}±1075	821.5~1148	993.7^{ab}±163.9	2.94~371.4	132.8^{b}±125.5
Phenyl acetaldehyde	177.7~272.7	210.1^{a}±43.61	175.3~257.9	210.2^{a}±31.38	38.23~125.5	72.71^{b}±39.68	203.6~282.5	235.4^{a}±41.64	68.34~234.6	127.2^{b}±52.78
2-Phenyl-2-butenal	207.6~326.8	248.9^{ab}±53.06	161.1~955.2	511.2^{a}±361.5	72.48~188	109^{b}±53.16	114.2~642.3	313.1^{ab}±287.1	10.82~453.2	89.72^{b}±142.3
Diacetyl	39.72~82.07	58.45^{a}±19.53	32.01~90.03	54.16^{a}±23.64	43.84~164.2	94.77^{a}±56.56	5.48~203.03	73.01^{a}±112.6	21.42~389.3	147.16^{a}±121.8
Glyoxal	1444~1655	1540^{a}±86.56	594.8~2124	1420^{a}±642.1	523.4~1609	1110^{a}±551.9	525.2~1722	1259^{a}±642.9	571.2~2371	1592^{a}±609
Methyl glyoxal	831.5~1166	956.9^{a}±153.7	290.2~1099	675.3^{a}±341.7	452.5~729.5	608.6^{a}±115.4	347.1~1213	645.5^{a}±491.4	353.5~6434	2323^{a}±2259
2-Butanone*	227.4~1396	603.4^{a}±538.1	133.6~1554	760.8^{a}±550	309.1~851.2	558.4^{a}±236.1	184~1080	535.^{a}±478.1	53.55~403.1	209.9^{a}±117.5
3-Methyl-2-butanone	4.11~20.1	8.39^{ab}±7.82	1.09~11.41	5.92^{ab}±3.99	4.99~9.61	7.36^{ab}±2.16	2.95~20.29	9.08^{a}±9.72	0.27~2.65	1.41^{b}±0.56
2-Pentanone	4.33~5.34	4.72^{a}±0.44	4.03~4.93	4.44^{a}±0.36	5.26~8.93	7.07^{a}±1.56	4.29~4.45	4.39^{a}±0.09	3.73~11.55	5.87^{a}±2.64
3-Pentanone	3.24~4.36	3.77^{a}±0.59	0~11.55	5.23^{a}±4.3	0.28~5.84	3.18^{a}±2.42	nq~9.99	3.38^{a}±5.73	1.74~7.98	4.04^{a}±2.12
2-Hexanone	1.01~2.53	1.71^{a}±0.66	0.96~2.36	1.64^{a}±0.55	1.61~1.77	1.66^{a}±0.07	0.36~1.21	0.7^{a}±0.45	0.19~2.64	0.81^{a}±0.81
4-Heptanone	2.14~4.87	3.17^{ab}±1.26	1.56~5.9	3.09^{ab}±1.47	3.9~8.42	5.26^{a}±2.12	0.49~1.43	0.88^{b}±0.49	0.4~5.69	2.15^{b}±2.14
2-Heptanone	1.76~5.1	3.33^{a}±1.53	3.99~8.02	5.64^{a}±1.6	2.23~5.72	3.33^{a}±1.61	1.75~13.15	5.85^{a}±6.33	0.7~5.46	2.26^{a}±1.44
2-Octanone	0.43~1.31	0.88^{a}±0.36	0.46~0.97	0.71^{ab}±0.2	0.51~0.69	0.6^{ab}±0.1	0.13~0.61	0.29^{b}±0.27	0.22~1.17	0.58^{ab}±0.31
2-Nonanone	5.32~13.25	8.97^{a}±3.32	4.83~10.74	7.81^{a}±2.68	0.6~8.49	4.56^{ab}±3.3	1.87~16.54	7.14^{a}±8.16	nd~3.68	1.03^{b}±1.27
2-Decanone	0.2~0.26	0.23^{a}±0.03	0.2~0.24	0.22^{ab}±0.02	0.18~0.22	0.2^{ab}±0.02	0.19~0.23	0.21^{ab}±0.02	nd~0.29	0.09^{b}±0.12
2-Furfural	13005~21347	17128^{a}±3846	4187~19885	11646^{a}±6165	6090~15472	10341^{a}±4119	7315~11530	8771^{a}±2390	1363~19676	12609^{a}±6072
5-Methyl-2-furfural	52.2~76.74	66.52^{b}±11.6	56.35~207.6	119.2^{ab}±59.83	41.61~76.45	51.27^{b}±16.81	66.5~201.3	145.4^{a}±70.29	44.72~179.8	108.7^{ab}±40.06
3-Pentene-2-one	747.3~1889	1141^{a}±519.9	270.4~1199	734.9^{a}±354.1	532.9~2491	1246^{a}±859.6	533.7~1001	770.3^{a}±233.9	301.1~2870	1568^{a}±838.5
1-Octen-3- one	0.07~0.08	0.08^{a}±0	0.07~0.11	0.08^{a}±0.01	0.08~0.09	0.08^{a}±0.01	nd~0.12	0.07^{a}±0.06	nd~0.25	0.09^{a}±0.08
6-Methyl-5-hepten-2-one	0.2~0.9	0.54^{a}±0.31	0.36~0.92	0.48^{a}±0.22	0.3~0.96	0.52^{a}±0.3	0.28~0.79	0.53^{a}±0.26	0.22~2	0.86^{a}±0.55
2-Cyclohexen-1-one	0.03~0.05	0.04^{a}±0.01	0.02~0.09	0.05^{a}±0.02	0.01~0.03	0.02^{a}±0.01	nd~0.1	0.03^{a}±0.06	0~2.88	0.36^{a}±0.85
Cyclopentanone	1.41~4.3	2.41^{b}±1.32	1.77~3.41	2.62^{b}±0.68	1.61~5.24	4.05^{ab}±1.65	2.54~4.18	3.42^{ab}±0.82	2.1~23.43	10.92^{a}±7.05
Cyclohexanone	5.58~7.48	6.44^{a}±0.98	5.1~7.14	6.25^{a}±0.8	5.31~6.3	5.82^{a}±0.53	4.4~7.47	6.21^{a}±1.61	5.05~17.6	7.42^{a}±3.58
Acetoin	23411~89406	56128^{ab}±31504	501.4~3917	2113^{b}±1319	19750~345311	123196^{a}±152457	15419~79871	52507^{ab}±33308	3220~47335	16602^{b}±12978

Acetophenone	29.0~117.3	79.8[ab]±38.7	21.0~84.0	34.7[b]±24.3	11.0~72.1	33.8[b]±26.9	30.4~154.5	102.8[a]±64.6	11.4~114.1	58.9[ab]±31.2
2-Phenyl-2-butenal	207.6~326.8	248.9[ab]±53.06	161.1~955.2	511.2[a]±361.5	72.48~188	109[b]±53.16	114.2~642.3	313.1[ab]±287.1	10.82~453.2	89.72[b]±142.3

* area/area of internal standard.

nq: not quantified, nd: not detected.
